# Sirtuins in neurodegenerative diseases: an update on potential mechanisms

**DOI:** 10.3389/fnagi.2013.00053

**Published:** 2013-09-25

**Authors:** Sang-Won Min, Peter D. Sohn, Seo-Hyun Cho, Raymond A. Swanson, Li Gan

**Affiliations:** ^1^Gladstone Institute of Neurological Disease, University of California at San FranciscoSan Francisco, CA, USA; ^2^Department of Neurology, University of California at San FranciscoSan Francisco, CA, USA; ^3^Neuroscience Graduate Program, University of California at San FranciscoSan Francisco, CA, USA; ^4^Veterans Affairs Medical Center, University of California at San FranciscoSan Francisco, CA, USA

**Keywords:** SIRT1, neurodegeneration, amyloid-β, tau, inflammation, NF-κB, mitochondria, epigenetic regulation

## Abstract

Silent information regulator 2 proteins (sirtuins or SIRTs) are a group of deacetylases (or deacylases) whose activities are dependent on and regulated by nicotinamide adenine dinucleotide (NAD^+^). Compelling evidence supports that sirtuins play major roles in many aspects of physiology, especially in pathways related to aging – the predominant and unifying risk factor for neurodegenerative diseases. In this review, we highlight the molecular mechanisms underlying the protective effects of sirtuins in neurodegenerative diseases, focusing on protein homeostasis, neural plasticity, mitochondrial function, and sustained chronic inflammation. We will also examine the potential and challenges of targeting sirtuin pathways to block these pathogenic pathways.

## INTRODUCTION

The past decade has seen an explosion of studies of sirtuins in health and disease. The founding member of the sirtuin gene family Sir2 was originally found in yeast, but others have since been found in many organisms (Kaeberlein et al., 1999). Mammalian sirtuins have seven homologs (SIRT1–7) that share a conserved catalytic core domain as class III histone deacetylases (HDACs) but exhibit different enzymatic activities in distinct subcellular localizations. Unlike class I and II HDACs, which require only zinc as a cofactor, sirtuins depend on NAD^+^ for activity. In the presence of NAD^+^, sirtuins catalyze the conversion of an acetylated substrate to a deacetylated substrate with *O*-acetyl-ADP-ribose and nicotinamide as side products.

SIRT1 shares the greatest homology with yeast Sir2 (Frye, 2000). In addition to histones, SIRT1 catalyzes deacetylation of a large number of non-histone substrates in the nucleus and cytoplasm. It is involved in diverse cellular functions, including maintaining genomic stability, suppressing inflammation, enhancing synaptic plasticity, and protecting against neurodegeneration. Like SIRT1, SIRT2 is a strong deacetylase with some common substrates in the cytoplasm and nucleus (Donmez and Outeiro, 2013). SIRT3–5 are localized mainly in the mitochondria. SIRT3 is the major mitochondrial deacetylase with a broad range of substrates (Newman et al., 2012). SIRT4 is a mitochondrial ADP-ribosyltransferase without any recognized deacetylase activity, and SIRT5 is both an NAD^+^-dependent deacetylase and deacylase (demalonylase and desuccinylase; Du et al., 2011). Localized exclusively in the nucleus, SIRT6 is a chromatin-bound NAD^+^-dependent deacetylase and an ADP-ribosyltransferase. SIRT7 is localized to the nucleolus and regulates ribosomal DNA gene expression (Ford et al., 2006; Jia et al., 2012; **Table 1**).

**Table 1:** Subcellular localizations and catalytic activities of mammalian sirtuins.

	Subcellular localization	Catalytic activity
SIRT1	Nucleus, cytoplasm	Deacetylase
SIRT2	Nucleus, cytoplasm	Deacetylase
SIRT3	Mitochondria	Deacetylase
SIRT4	Mitochondria	ADP-ribosyltransferase
SIRT5	Mitochondria	Deacetylase, deacylase
SIRT6	Nucleus	Deacetylase, ADP-ribosyltransferase
SIRT7	Nucleolus	Deacetylase

Earlier studies showed that the lifespan of yeast was shortened by deleting Sir2, but lengthened by its overexpression (Kaeberlein et al., 1999). Increased dosages of the Sir2 orthologs, SIR-2.1 and dSIR2, also extended lifespan of worm (Tissenbaum and Guarente, 2001) and fly (Rogina and Helfand, 2004). Most recently, the anti-aging role of Sir2 has been challenged and refined (Burnett et al., 2011; Viswanathan and Guarente, 2011; Banerjee et al., 2012). Nevertheless, compelling evidence supports a major role for sirtuins in many aspects of physiology from protozoa to metazoan (Michan and Sinclair, 2007; Baur et al., 2012; Libert and Guarente, 2013).

This review focuses on our current understanding of the role of sirtuins in several instrumental pathogenic processes in neurodegenerative diseases, with special emphasis on protein homeostasis, neural plasticity, mitochondrial function, and inflammatory pathways.

## PROTEIN HOMEOSTASIS

Neurodegenerative diseases have distinct clinical manifestations, mostly due to the impairment of specific neural networks. However, they share key features, one of which is loss of protein homeostasis in the form of intra- or extracellular accumulation of soluble and insoluble protein aggregates. These protein aggregates are hallmarks of the disease and include amyloid β (Aβ), hyperphosphorylated tau, cytosolic TDP-43, α-synuclein, huntingtin, and others.

**FIGURE 1:**
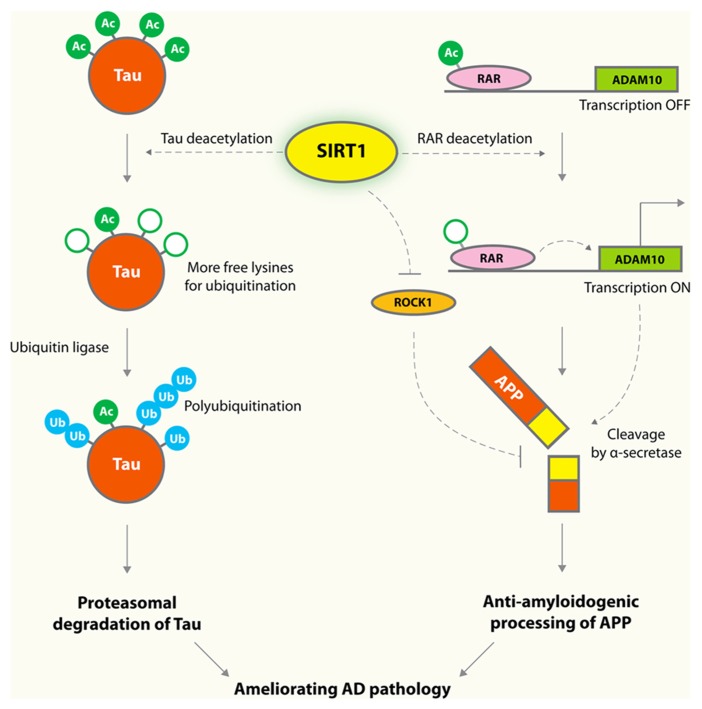
**Protective mechanisms of SIRT1 in AD.** SIRT1 deacetylates tau protein at multiple residues, and enhances tau polyubiquitination and subsequent proteasomal degradation. Overexpression of SIRT1 deacetylates retinoic acid receptor (RAR) β and activates ADAM10, a component of the α-secretase, which processes APP along an anti-amyloidogenic pathway that decreases formation of toxic Aβ species. SIRT1 was also shown to enhance α-secretase cleavage via a mechanism involving reducing ROCK1, which suppresses α-secretase cleavage.

Alzheimer’s disease (AD) is the most common dementia in the elderly population. Aβ peptides are believed to be a key culprit in AD (Hardy and Selkoe, 2002). Although amyloid plaques are well-recognized pathological hallmarks in AD brains, specific types and forms of soluble Aβ oligomers have been implicated as the earliest triggers in the amyloid toxicity pathway (Klein, 2002; Lesne et al., 2006; Querfurth and LaFerla, 2010). The amyloid hypothesis is strongly supported by human genetic analyses: the vast majority of familial mutations that cause early-onset AD are associated with increased production of Aβ peptides, leading to imbalance in homeostatic control of protein levels (Tanzi and Bertram, 2005). In contrast, a coding mutation that protects against AD and cognitive decline also reduces the production of Aβ by 40% (Jonsson et al., 2013). SIRT1 lowers Aβ levels by reducing its production from amyloid precursor protein (APP). Cleavage of APP by α-secretase results in production of non-amyloidogenic fragments, and cleavage by β- and γ-secretases results in various Aβ and C-terminal fragments (Hardy and Selkoe, 2002). In cultured cells, SIRT1 enhances α-secretase activity via inhibiting the Rho-associated, coiled-coil-containing protein kinase 1 (ROCK), and reduces Aβ production (**Figure 1**; Qin et al., 2006). A more recent study showed that SIRT1 also enhances the transcription of ADAM10, a member of α-secretase family, by deacetylating and activating the retinoic acid receptor β (Donmez et al., 2010; **Figure 1**).

Hyperphosphorylated tau accumulates in the form of neurofibrillary tangles in neurodegenerative tauopathies, including AD and frontotemporal lobar degeneration with tau-positive inclusions (FTLD-tau; Cairns et al., 2007; Ludolph et al., 2009). In AD, the number and extent of neurofibrillary tangles are the best pathological hallmark correlating with the severity of cognitive impairments and neurodegenerations whereas the plaque loads do not correlate at all with regional atrophy and synaptic loss (Berg et al., 1998). SIRT1 levels correlate negatively with the amount of neurofibrillary tangles in AD brains (Julien et al., 2009), and this association suggests a possible pathogenic role of SIRT1 deficiency in tau accumulation. Indeed, tau was acetylated in the early and late stages of tauopathies (Min et al., 2010; Cohen et al., 2011; Irwin et al., 2012; Grinberg et al., 2013). Aberrant tau acetylation has a key role in tau accumulation and dysfunction (Min et al., 2010; Cohen et al., 2011; Irwin et al., 2012; Grinberg et al., 2013). SIRT1 deacetylates tau, and its deficiency results in enhanced levels of acetylated tau and phosphorylated tau (Min et al., 2010; **Figure 1**).

α-Synuclein has been implicated in several neurodegenerative diseases, including dementia with Lewy bodies, Parkinson’s disease (PD), and multiple system atrophy (Goedert et al., 2013). SIRT1 suppresses the formation of α-synuclein inclusions in *Caenorhabditis elegans* (van Ham et al., 2008). Treatment with resveratrol, an indirect activator of SIRT1, protected cells of the human neuroblastoma line SK-N-BE against toxicity induced by expression of α-synuclein carrying familial PD mutation (A30P; Albani et al., 2009). This protective mechanism depends on SIRT1; treatment with sirtinol, a specific inhibitor of SIRT1, restored toxicity. SIRT1 reduced the number of α-synuclein aggregates in the brains of mice expressing A53T α-synuclein. SIRT1 deacetylates heat shock factor-1 (Westerheide et al., 2009), resulting in an increased expression of Hsp70, a molecular chaperon that could serve as a protective mechanism against α-synuclein toxicity (Donmez et al., 2012). In contrast, inhibiting SIRT2 rescued α-synuclein-mediated toxicity and modified aggregation in models of PD *in vitro* (Outeiro et al., 2007). The opposing effects of SIRT1 and SIRT2 on synucleinopathies could reflect their distinct subcellular localizations and substrates.

In mammalian cells, misfolded proteins can be removed by the proteasome or the autophagy–lysosomal pathways. Since acetylation and ubiquitination both occur at lysine residues, acetylation often interferes with polyubiquitination, which is required for proteasome-mediated degradation. Thus, lack of SIRT1 induces hyperacetylation of the substrate proteins, which preclude them from the polyubiquitination process, resulting in increased steady-state protein levels. For example, inhibition of SIRT1 blocks tau polyubiquitination and tau turnover, likely via increased acetylation of tau on lysine residues that are also subject to polyubiquitination (Min et al., 2010).

SIRT1 deacetylates autophagy gene products and stimulates basal rates of autophagy (Lee et al., 2008), which has emerged as an important route for the removal of toxic misfolded protein aggregates that accumulate in neurodegenerative diseases (Levine and Kroemer, 2008). Autophagy induced by SIRT1 activation prevented neurotoxicity by prion protein fragment (106–126) in a neuronal cell line (Jeong et al., 2013). Degradation of α-synuclein was also enhanced by SIRT1 activator via autophagy induction in α-synuclein-expressing PC12 cell lines (Wu et al., 2011). In agreement with these findings in mammalian cells, Sir2 promotes both autophagy and mitophagy in *Saccharomyces cerevisiae* (Sampaio-Marques et al., 2012). In contrast to the autophagy-enhancing effects of SIRT1, SIRT2 inhibits the autophagy-mediated degradation of protein aggregates in neuronal cell lines (Gal et al., 2012). In a neuronal cell line, overexpression of SIRT2 inhibits lysosome-mediated autophagic turnover of protein aggregates and exacerbates toxicity induced by Aβ (Gal et al., 2012).

## NEURONAL PLASTICITY

Regulation of the formation and maintenance of memory involves epigenetic mechanisms, such as post-translational modifications of histone tails, DNA methylation, and non-coding RNA (Fischer et al., 2007; Day and Sweatt, 2011; Wang et al., 2012). Brain-specific SIRT1 knockout mice showed deficits in learning and memory, supporting the importance of SIRT1 in maintaining neural plasticity (Gao et al., 2010). Whether and how other sirtuins might regulate neural plasticity remains to be determined.

Brain-derived neurotrophic factor (BDNF), which plays a critical role in neural plasticity (Lipsky and Marini, 2007), is enhanced by SIRT1 (Gao et al., 2010). Specifically, it increases the number of dendritic spines, neuronal connectivity, and memory function. SIRT1 deficiency reduces BDNF expression by upregulating the microRNA miR-134 (Gao et al., 2010). SIRT1 forms a repressor complex with the transcription factor YY1 to suppress miR-134 expression (Gao et al., 2010). Another mechanism by which SIRT1 regulates BDNF involves deacetylation of methyl-CpG binding protein 2 (MeCP2). This action allows MeCP2 to be released from the methylated CpG sites within the BDNF exon 4 promoter, resulting in increased BDNF transcription in hippocampus (Zocchi and Sassone-Corsi, 2012).

The importance of cAMP response element-binding protein (CREB) as a crucial regulator for learning and memory process is conserved from mollusk neurons in culture to complex behaviors in mammals (Bliss and Collingridge, 1993; Alberini et al., 1994). Like BDNF, SIRT1 enhances CREB expression through the miR-134 pathway (Gao et al., 2010). SIRT1 directly deacetylates CREB and modulates its activity in liver (Qiang et al., 2011) but not in brain (Fusco et al., 2012). CREB is involved in the brain’s response to CR, which upregulates SIRT1 levels. Increased SIRT1 levels, in turn, enhance CREB-dependent expression of genes involved in neuronal metabolism, survival, and plasticity (Fusco et al., 2012). Although the exact molecular mechanism underlying the CREB–SIRT1 axis is unknown, these findings highlight a unique molecular network at the crossroad of energy metabolism, metabolic diseases, and brain aging.

## MITOCHONDRIAL FUNCTIONS

Mitochondria are critical regulators of neuronal survival and death. They produce energy in response to nutrient availability and are the main contributors of oxidative stress. Accumulating lines of evidence suggest that disruption of mitochondrial processes leads to neurodegenerative diseases (Lin and Beal, 2006). In a proteomic survey of lysine acetylation, more than 20% of mitochondrial proteins are acetylated on their lysine residues (Newman et al., 2012). This study supports the importance of modulatory role of sirtuins as deacetylases in the maintenance of mitochondrial functions. Most studies focused on SIRT1 and SIRT3 as the primary regulators of mitochondrial biology via deacetylation (Brenmoehl and Hoeflich, 2013). SIRT4 and SIRT5 are also localized in mitochondria. However, SIRT4 has only weak ADP ribosyltransferase activity (Verdin et al., 2010), and SIRT5 regulates malonylation and succinylation (Du et al., 2011). Indeed, SIRT3 knockout mice display remarkable increases in the acetylation levels of mitochondrial proteins, whereas no mitochondrial hyperacetylation was observed when the two other mitochondrial sirtuins, SIRT4 and SIRT5, were deleted (Lombard et al., 2007).

SIRT1 deacetylates and activates PGC-1α, a transcriptional coactivator that regulates several key mitochondrial processes, including mitochondrial biogenesis and oxidative phosphorylation (Rodgers et al., 2005). SIRT1 also enhances transcription of *PGC-1α* gene via interaction with MyoD, which binds to the PGC-1α promoter (Amat et al., 2009). PGC-1α is required for the induction of reactive oxygen species (ROS)-detoxifying enzymes. PGC-1α null mice show high degree of sensitivity to the neurodegenerative effects of oxidative stressors (St-Pierre et al., 2006). In models of Huntington’s disease (HD), transcription of PGC-1α is repressed by mutant huntingtin, the protein that causes HD. PGC-1α knockout exacerbates neurodegeneration and motor abnormalities in the HD knockin mice. Conversely, PGC-1α expression ameliorates, in part at least, mitochondrial dysfunction and neuronal toxicity induced by mutant huntingtin (Cui et al., 2006). Elevating SIRT1 activity ameliorated neuronal dysfunction induced by mutant polyglutamines in *C. elegans* (Jiang et al., 2012). Polyglutamine cytotoxicity is prevented by SIRT1 activation in neurons derived from HdhQ111 KI mice (Parker et al., 2005). In mouse models of HD, ablation of SIRT1 exacerbates neurodegeneration (Jeong et al., 2012), whereas SIRT1 overexpression improves motor functions and brain atrophy (Jiang et al., 2012). Thus, SIRT1 protects against HD-related neurodegeneration at least partially by preventing mitochondrial impairment through PGC-1α activation.

SIRT3 resides in the mitochondrial matrix and regulates the acetylation of a number of metabolic enzymes in mitochondria (Qiu et al., 2010). Because neurons have high demand in cellular energy and limited regenerative capacity, mitochondrial adaptation to changes in energy availability is a critical strategy that protects neurons from degeneration (Andersen, 2004). SIRT3 expression is responsive to changes in nutrient levels, and its upregulation results in deacetylation of enzymes involved in metabolic pathways that utilize alternative energy sources such as fatty acids (Hynd et al., 2004; Someya et al., 2010). Pathogenic protein accumulation can occur in mitochondria in AD neurons, thereby disrupting mitochondrial electron transport chain and increasing ROS production (Manczak et al., 2006). Increased indices of ROS damage have been found in affected brain regions of neurodegenerative diseases such as AD and PD (Andersen, 2004). SIRT3 deacetylates and increases activity of the superoxide dismutase SOD2 that reduces oxidative stress (Qiu et al., 2010). A protective effect of SIRT3 against oxidative stress also contributes to caloric restriction (CR)-mediated suppression of degeneration of cochlear neurons (Someya et al., 2010). Mechanistically, SIRT3 deacetylates and activates mitochondrial isocitrate dehydrogenase 2, leading to increased levels of nicotinamide adenine dinucleotide phosphate (NADPH), which in turn elevated levels of reduced glutathione in mitochondria (Someya et al., 2010). Mitochondrial SIRT3 is also regulated by various stresses in neurodegenerative diseases. SIRT3 is upregulated by elevated levels of ROS during excitotoxic stress (Hynd et al., 2004), a common feature of neurodegenerative diseases, such AD. Upregulation of SIRT3 protected against excitotoxicity (Kim et al., 2011). SIRT3 is also upregulated when ROS is pharmacologically augmented in neuronal culture and in human AD brains (Weir et al., 2012). Interestingly, SIRT3 levels were reduced in cells expressing mutant huntingtin (Fu et al., 2012). SIRT3 upregulation mediated by small molecules decreased levels of ROS and prevented mitochondrial dysfunction and cytotoxicity induced by mutant huntingtin (Fu et al., 2012).

## INFLAMMATORY RESPONSES

In aging, genes involved in inflammatory responses are upregulated in the human brain (Lu et al., 2004; Franceschi et al., 2007; Krstic and Knuesel, 2013). On the other hand, CR, which activates sirtuin pathways, attenuates aging-induced upregulation of inflammatory genes (Cao et al., 2001). These findings highlight an intriguing connection between the anti-inflammatory function of sirtuins and their potent anti-aging effects. In addition, many age-related neurodegenerative diseases, such as AD, frontotemporal dementias (FTD), amyotrophic lateral sclerosis (ALS), and PD, exhibit prolonged inflammatory responses (Glass et al., 2010). Thus, anti-inflammatory effects of sirtuins could have broad relevance in aging and neurodegeneration.

Nuclear factor kappa B (NF-κB) is a master regulator of immune response and inflammation (Hayden and Ghosh, 2012). Bioinformatics analyses identified the NF-κB binding domain as the motif most strongly associated with the aging process (Adler et al., 2007). In the skin of aged mice, genetic blockade of NF-κB reversed the global gene expression program and tissue characteristics to those of young mice (Adler et al., 2008). The mechanism might involve activation of redox-sensitive transcription factors by the cumulative effects of oxidative damage during aging. For example, the increased production of ROS during aging is associated with upregulation of NF-κB (Kabe et al., 2005). Activation of NF-κB, in turn, induces the expression of proinflammatory genes, including cytokines, growth factors, and chemokines (Mattson and Meffert, 2006). Since some of the NF-κB-induced proteins are also potent NF-κB activators, the resulting vicious cycle may contribute to establishment of a chronic inflammatory state and related pathologies.

NF-κB is also important in modulating cellular senescence. Genetic reduction of p65 subunit or pharmacological inhibition of NF-κB delayed the onset of progeroid symptoms in accelerated aging mouse model caused by a defect in DNA repair (Tilstra et al., 2012). In human fibroblasts, senescence induced by activation of p16 and p53 resulted in global regulation of NF-κB pathways; silencing of NF-κB overcame senescence (Rovillain et al., 2011). Senescence is also suppressed by overexpressing SIRT1, which was downregulated in the senescent cells (Rovillain et al., 2011). Thus, the beneficial effects of SIRT1 on senescence are likely mediated by inactivation of NF-κB pathways.

**FIGURE 2:**
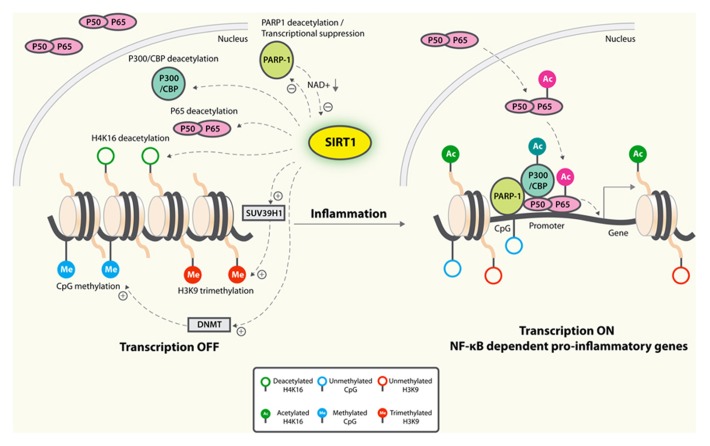
**Anti-inflammatory mechanisms of SIRT1.** SIRT1 deacetylates p65 and blocks the transactivation of NF-κB-dependent gene expression. SIRT1 suppresses the activity of PARP-1, a coactivator of NF-κB-dependent transcription, by deacetylation and by inhibiting its expression. PARP-1 activation could deplete NAD^+^, resulting in inhibition of SIRT1 and NF-κB activation. On the epigenetic level, SIRT1 represses NF-κB-dependent inflammatory gene expression by deacetylating H4K16 and also by recruiting more components of repressor complexes. SIRT1 deacetylates and activates histone methyltransferase SUV39H1, which suppresses expression of inducible inflammatory genes. DNA methylation is associated with suppressed expression. Whether SIRT1 could inhibit expression of inflammatory genes by enhancing promoter methylation remains to be determined.

NF-κB-dependent transcription is repressed by SIRT1, which deacetylates p65/RelA at lysine 310 (Yeung et al., 2004; **Figure 2**). Macrophages derived from myeloid cell-specific SIRT1 knockout mouse model had higher levels of proinflammatory cytokines that are associated with NF-κB hyperacetylation (Schug et al., 2010). In primary cortical cultures, microglial NF-κB activation played a critical role in Aβ-mediated neuronal death (Chen et al., 2005). Increased expression of SIRT1 or treatment with resveratrol markedly reduced Aβ-dependent NF-κB activation in microglia and neuronal loss, suggesting that SIRT1 blocks the neuropathogenic inflammatory loops (Chen et al., 2005; **Figure 2**).

NF-κB-dependent gene activation is strongly modulated by poly(ADP-ribose) polymerase-1 (PARP-1), which possesses a DNA binding domain enabling them to bind to damaged DNA and thus become activated (Beneke, 2012). It consumes NAD^+^ in the process of forming (ADP-ribose) polymers on target proteins (Canto et al., 2013). In cardiomyocytes, SIRT1 interacts and deacetylates PARP-1, resulting in reduced PARP-1 activity (Rajamohan et al., 2009). SIRT1 is also capable of suppressing the activity of the PARP-1 gene promoter, leading to decreased PARP-1 protein synthesis (Rajamohan et al., 2009). As PARP-1 is required and sufficient for specific transcriptional activation of NF-κB, it is thus conceivable that SIRT1 could suppress NF-κB signaling by reducing PARP-1 (**Figure 2**). Further studies will be needed to establish the causative link. On the other hand, since PARP-1 uses NAD^+^ as a substrate to synthesize PAR, excessive PARP-1 activation could deplete NAD^+^ levels, resulting in SIRT1 inhibition and NF-κB activation (**Figure 2**). In primary astrocyte cultures, activation of PARP-1 with *N*-methyl-*N*′-nitro-*N*-nitrosoguanidine resulted in sustained acetylation of p65 and NF-κB activation, likely by inhibiting SIRT1 due to depleted NAD^+^ levels (Kauppinen et al., 2013). Consistent with this notion, addition of exogenous NAD^+^ led to p65 deacetylation and inhibition of NF-κB signaling (Kauppinen et al., 2013). In contrast, inhibition of basal PARP-1 activity in myotubes and in muscle raised NAD^+^ levels and enhanced SIRT1 activity. This correlative evidence supports that the increased NAD^+^ availability might be a key mechanism by which PARP deficiency activates SIRT1 (Bai et al., 2011). However, more studies are needed to establish direct regulation of SIRT1 activity by the relatively small magnitude changes in cytosolic NAD concentrations that occur under physiological conditions.

Inflammatory responses are regulated by epigenetic changes (Medzhitov and Horng, 2009), which are defined as mitotically and meiotically heritable changes in gene function that do not depend on DNA sequence (Bernstein et al., 2007). SIRT1 deacetylates and inactivates the transactivation state of p65, deacetylates specific lysines on histone H3 and H4, H1 nucleosome linker, and histone methyltransferase suppressor of variegation 3–9 homolog 1 (SUV39H1); and modulates activities of DNA CpG methyltransferases (McCall et al., 2011). Additionally, SIRT1 could deacetylate and inactivate p300/CBP, critical coactivators of NF-κB-dependent expression of inflammatory genes (Chen and Greene, 2003). A number of epigenetic mechanisms are involved in SIRT1-mediated regulation of inflammatory responses (**Figure 2**).

### HISTONE ACETYLATION

SIRT1 might repress inflammatory responses by combining deacetylation of histones and recruitment of non-histone proteins, such as p65/RelA (Liu et al., 2011). During endotoxin tolerance when transition from hyperinflammation to hypoinflammation occurs, SIRT1 deacetylates H4K16 to terminate NF-κB-dependent transcription. SIRT1 represses gene expression by deacetylating histones and also by remaining bound to TNFα and IL-1β promoter regions to recruit more components of repressor complexes, such as histone H1, RelB, and methyltransferases (Liu et al., 2011; **Figure 2**).

### HISTONE METHYLATION

Unlike histone acetylation, which is associated with active transcription, histone methylation is often associated with transcriptional repression (Bernstein et al., 2007). SIRT1 could suppress expression of inflammatory genes by enhancing the activities of histone methyltransferases. For example, SIRT1 deacetylates and activates histone methyltransferase SUV39H1, resulting in increased levels of the trimethyl H3K9 (Vaquero et al., 2007), which suppresses expression of inducible inflammatory genes (Saccani and Natoli, 2002; **Figure 2**).

### DNA METHYLATION

Inflammatory gene expression could be regulated by methylation of CpG sites on the promoter region, which is often associated with transcriptional repression (Hartnett and Egan, 2012). For example, DNA methylation of IL1β inversely correlated with the levels of mRNA (Hashimoto et al., 2013). Since SIRT1 also modulates the activities of DNA methyltransferases (Peng et al., 2011), it remains to be determined if SIRT1 suppresses inflammatory responses via increasing methylation of promoter regions of inflammatory genes (**Figure 2**).

Hyperactive NF-κB signaling in SIRT6-deficient mice is important for the premature aging phenotype (Kawahara et al., 2009). In SIRT6-deficient cells, hyperacetylation of H3K9 at these target promoters is associated with increased RelA promoter occupancy and enhanced NF-κB-dependent modulation of gene expression, apoptosis, and cellular senescence (Kawahara et al., 2009). Partial inactivation of NF-κB ameliorated the premature aging phenotype in SIRT6 KO mice (Kawahara et al., 2009). Thus, continued NF-κB activation is required to enforce many features of aging. Interestingly, overexpression of SIRT6 in male mice, but not in female mice, resulted in significantly longer lifespan than wild-type mice (Kanfi et al., 2012). Male mice overexpressing SIRT6 had lower serum levels of insulin-like growth factor 1 (IGF1), a key pathway for regulating lifespan (Kenyon, 2010). Thus, SIRT6 could promote longevity via multiple downstream signaling pathways.

## CONCLUSION AND THERAPEUTIC PERSPECTIVES

Sirtuins block multiple key processes in neurodegeneration. They restore protein homeostasis by reducing accumulation of toxic protein aggregates, improve neural plasticity by elevating transcription of genes important for learning and memory, enhance mitochondria function by reducing oxidative stress, and suppress sustained chronic inflammation via inhibiting NF-κB combined with epigenetic mechanisms.

However, it is important to recognize that the effects and regulation of sirtuins are extremely complex. Broad activation of sirtuins will lead to deacetylation of histones and various non-histone proteins, which may affect diverse cellular functions. For example, SIRT1 and SIRT2 appear to have opposite effects on the aggregation of misfolded proteins. Activation of a given sirtuin may have divergent outcomes, depending on pathophysiological circumstances. Nevertheless, specific sirtuin modulators could have broad therapeutic potential against various neurodegenerative diseases.

There has been an intense debate surrounding the importance of sirtuin enzymes in mediating the effects of resveratrol and other small-molecule compounds that activate SIRT1-related pathways (see Baur et al., 2012 for a comprehensive review on this topic). More studies will be needed to resolve the discrepancies and to develop new SIRT1 activators that can pass the blood–brain barrier and improve CNS functions in models of neurodegenerative diseases.

## Conflict of Interest Statement

The authors declare that the research was conducted in the absence of any commercial or financial relationships that could be construed as a potential conflict of interest.
